# Oligo-fucoidan prevents renal tubulointerstitial fibrosis by inhibiting the CD44 signal pathway

**DOI:** 10.1038/srep40183

**Published:** 2017-01-18

**Authors:** Cheng-Hsien Chen, Yuh-Mou Sue, Chung-Yi Cheng, Yen-Cheng Chen, Chung-Te Liu, Yung-Ho Hsu, Pai-An Hwang, Nai-Jen Huang, Tso-Hsiao Chen

**Affiliations:** 1 Division of Nephrology, Department of Internal Medicine, Wan Fang Hospital, Taipei Medical University, Taipei, Taiwan; 2 Department of Internal Medicine, School of Medicine, College of Medicine, Taipei Medical University, Taipei, Taiwan; 3 Division of Nephrology, Department of Internal Medicine, Shuang Ho Hospital, Taipei Medical University, New Taipei City, Taiwan; 4 School of Medicine, National Defense Medical Center, Taipei, Taiwan; 5 Department of Bioscience and Biotechnology, National Taiwan Ocean University, Taiwan

## Abstract

Tubulointerstitial fibrosis is recognized as a key determinant of progressive chronic kidney disease (CKD). Fucoidan, a sulphated polysaccharide extracted from brown seaweed, exerts beneficial effects in some nephropathy models. The present study evaluated the inhibitory effect of oligo-fucoidan (800 Da) on renal tubulointerstitial fibrosis. We established a mouse CKD model by right nephrectomy with transient ischemic injury to the left kidney. Six weeks after the surgery, we fed the CKD mice oligo-fucoidan at 10, 20, and 100 mg/kg/d for 6 weeks and found that the oligo-fucoidan doses less than 100 mg/kg/d improved renal function and reduced renal tubulointerstitial fibrosis in CKD mice. Oligo-fucoidan also inhibited pressure-induced fibrotic responses and the expression of CD44, β-catenin, and TGF-β in rat renal tubular cells (NRK-52E). CD44 knockdown downregulated the expression of β-catenin and TGF-β in pressure-treated cells. Additional ligands for CD44 reduced the anti-fibrotic effect of oligo-fucoidan in NRK-52E cells. These data suggest that oligo-fucoidan at the particular dose prevents renal tubulointerstitial fibrosis in a CKD model. The anti-fibrotic effect of oligo-fucoidan may result from interfering with the interaction between CD44 and its extracellular ligands.

Chronic kidney disease (CKD) is a progressive illness that is becoming a global public health problem. Although the incidence and cost of CKD are increasing^1^, there are few effective pharmacological agents for preventing and treating CKD. CKD shares a common appearance with glomerulosclerosis, vascular sclerosis and tubulointerstitial fibrosis, suggesting a common final pathway of progressive injury^2^,^3^. Among these fibrotic progresses, tubulointerstitial fibrosis is recognized as a key determinant of progressive CKD due to the strong correlation between the degree of interstitial fibrosis and renal functional loss^4^. Tubulointerstitial fibrosis mainly involves interstitial myofibroblasts, which precede the accumulation of extracellular matrix, including fibronectins and collagens^1^. In the past decades many studies used unilateral ureteric obstruction in rodents as a model of renal fibrosis^5^. Sustained obstruction can convey pressure on the renal tubular system resulting in fibrosis^6^. Recent studies revealed that pressure increases the expression of transforming growth factor (TGF)-β, CD44, alpha smooth muscle actin (αSMA), fibronectin, and other fibrosis-related proteins in renal epithelial cells^7^,^8^,^9^. TGF-β is a profibrotic cytokine found in CKD that modulates fibrotic processes through a variety of signaling pathways, including the Smad and MAPK pathways^10^. CD44, a cell surface glycoprotein, is expressed on tubular epithelial cells solely upon kidney injury^11^,^12^. The interaction between CD44 and its ligands, hyaluronan and osteopontin, mediates pressure-induced fibrotic responses in renal tubular cells at the early stage^9^. Fibronectin is one of the extracellular matrix components that accumulate during renal fibrosis^10^,^13^. Increased αSMA is one of the major characteristics of mesenchymal cells that contribute to the pathogenesis of renal fibrosis^14^. Moreover, a subunit of the cadherin protein complex, β-catenin, acts as an intracellular signal transducer in the Wnt signaling pathway, which is also involved in initial mechanisms promoting renal fibrosis^15^,^16^,^17^. Understanding the initial mechanism of tubulointerstitial fibrosis is helpful for developing therapeutic strategies for CKD.

Fucoidan is a natural, fucose-enriched, sulphated polysaccharide found mainly in various species of brown algae and brown seaweed. The use of low molecular weight fucoidan (<7 kDa) is currently thought to be safe up to 2000 mg/kg body weight per day in rats^18^,^19^ and presents no significant genotoxic concern^20^. Fucoidan has been reported to have antiviral, antioxidant, antimicrobial, anticoagulant, antitumor, and anti-inflammatory properties^21^. Fucoidan is also reported to exert beneficial effects in some murine nephropathy models. Post ischemic kidneys may be protected against reperfusion injury by intravenous fucoidan administration at 10 mg/kg^22^. Up to 200 mg/kg body weight orally administered fucoidan reduced the symptom development of chronic renal failure or Heymann nephritis in a rat model^23^,^24^. However, the influence of fucoidan on renal fibrosis is not yet clear. In the present study, we investigated the influence of oligo-fucoidan (~800 Da) on renal tubulointerstitial fibrosis in CKD mice (right nephrectomy with transient ischemic injury to the left kidney) and its molecular mechanism by an *in vitro* study.

## Results

### Oligo-fucoidan improves renal function in CKD mice

**Figure 1:**
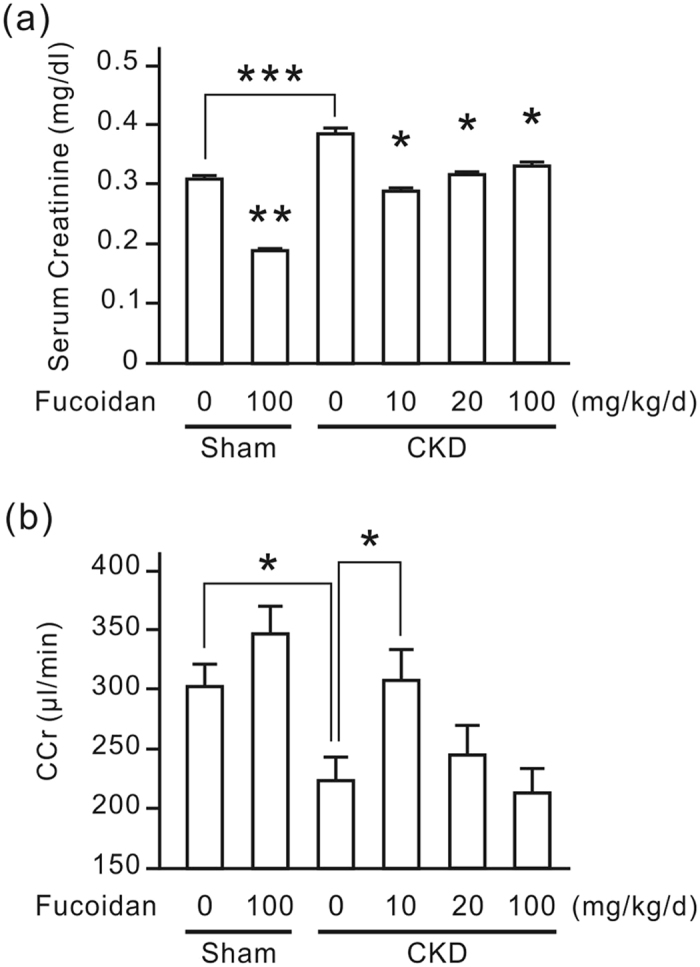
Oligo-fucoidan improves the renal function in CKD mice. Six weeks after the surgery, the experimental mice were fed with or without oligo-fucoidan at 10, 20, or 100 mg/kg/d for 6 weeks. The blood and the 24-h urine of each mouse were analyzed to detect renal function. (**a**) Serum creatinine. The results are expressed as the means ± SD from 6 mice in each group. *P < 0.05 vs. the CKD group without oligo-fucoidan. **P < 0.05 vs. the sham group without oligo-fucoidan. ***P < 0.05. (**b**) Creatinine clearance rate (CCr). The results are expressed as the means ± SD from 6 mice in each group. *P < 0.05.

To evaluate the influence of oligo-fucoidan on renal tubulointerstitial fibrosis *in vivo*, we established a CKD mouse model by right nephrectomy with transient ischemic injury to the left kidney. Six weeks after the surgery, we fed the experimental mice oligo-fucoidan for 6 weeks. The renal function of experimental mice was monitored by measuring serum creatinine and the creatinine clearance rate (CCr). Serum creatinine was significantly elevated in CKD mice, which was inhibited by oligo-fucoidan treatment (Fig. 1a). The inhibitory effect of oligo-fucoidan was strongest at the dosage of 10 mg/kg/d, and was reduced along with increasing dosage. In the sham group, oligo-fucoidan also significantly reduced serum creatinine. The CCr was significantly reduced in CKD mice, which recovered with oligo-fucoidan treatment at a dose of 10 mg/kg/d (Fig. 1b). The oligo-fucoidan dose of 100 mg/kg/d, however, did not improve the CCr in CKD mice. In the sham group, oligo-fucoidan a dose of 100 mg/kg/d significantly increased CCr. This result indicates that a low dosage of oligo-fucoidan improves renal function in CKD mice.

### Oligo-fucoidan inhibits renal tubulointerstitial fibrosis in CKD mice

**Figure 2:**
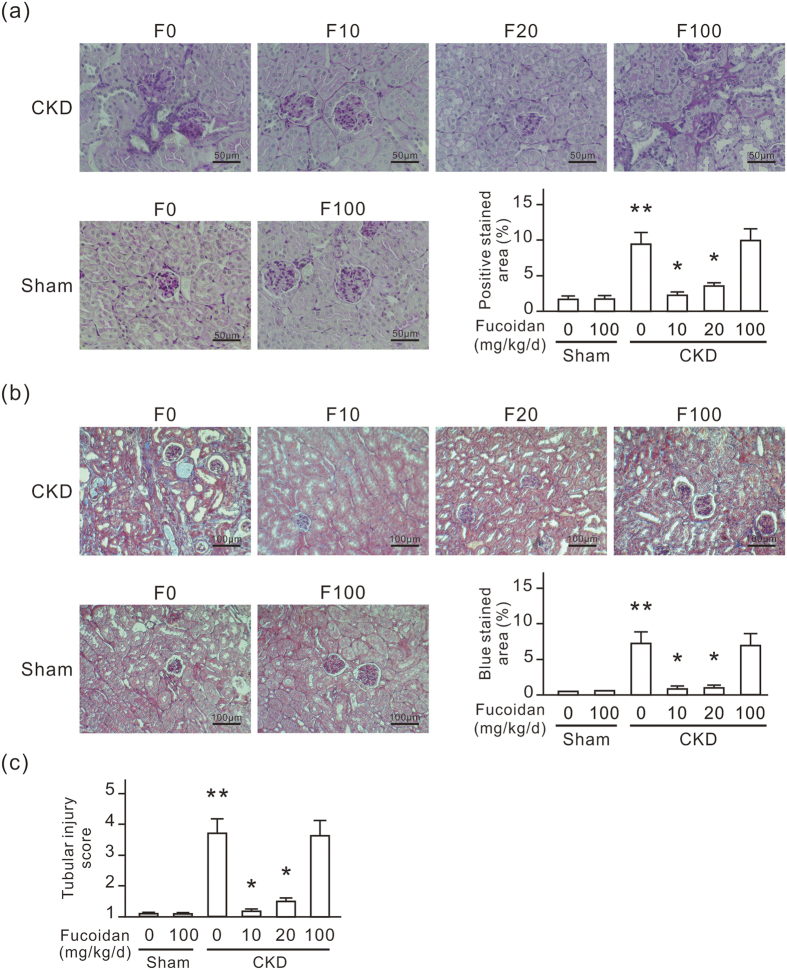
Oligo-fucoidan at low dosage inhibits renal tubulointerstitial fibrosis and tubular injury in CKD mice. Six weeks after the surgery, the experimental mice were fed with or without oligo-fucoidan at 10 (F10), 20 (F20), or 100 (F100) mg/kg/d for 6 weeks, and then the kidneys were collected. (**a**) PAS-stained paraffin-embedded mouse kidney sections. Scale bars = 50 μm. (**b**) Masson’s trichrome stained paraffin-embedded mouse kidney sections. Scale bars = 100 μm. (**c**) The tubular injury score. Magenta and blue areas in PAS and Masson’s trichrome stained slides respectively are also presented in a bar chart. Oligo-fucoidan treatment at a dose of 10 or 20 mg/kg/d significantly reduced PAS-positive matrix, collagen accumulation, tubular injury and renal tubule dilation in CKD mice, but not the treatment at 100 mg/kg/d. The data are shown as the means ± SD from 6 mice in each group. *P < 0.05 vs. the CKD group without oligo-fucoidan. **P < 0.05 vs. the sham group without oligo-fucoidan.

Periodic acid-Schiff (PAS)- and Masson’s trichrome-stained sections of kidneys were examined for histological evidence of renal damage. For 12 weeks following the operation, CKD mice accumulated PAS-positive matrix in the glomeruli and the tubulointerstitial spaces of the kidneys (Fig. 2a). Some renal tubules were dilated in CKD mice. Under Masson’s trichrome staining, collagen fibers appeared light blue. Progressively accumulation of collagen was considered as a hallmark of tissue fibrosis. In CKD mouse kidneys, a large amount of blue proliferating collagen around renal tubules was observed (Fig. 2b). The tubular injury score in the CKD group was greater than that in the Sham group (Fig. 2c). Oligo-fucoidan treatment at a dose of 10 or 20 mg/kg/d for 6 weeks significantly reduced PAS-positive matrix, collagen accumulation, tubular injury and renal tubule dilation in CKD mice. However, this protective effect did not occur with the oligo-fucoidan treatment at 100 mg/kg/d. In the sham group, oligo-fucoidan at a dose of 100 mg/kg/d did not cause any histological change in the kidneys. These results indicate that a low dosage of oligo-fucoidan inhibits renal tubulointerstitial fibrosis in CKD mice.

### Oligo-fucoidan reduces renal fibronectin and αSMA in CKD mice

**Figure 3:**
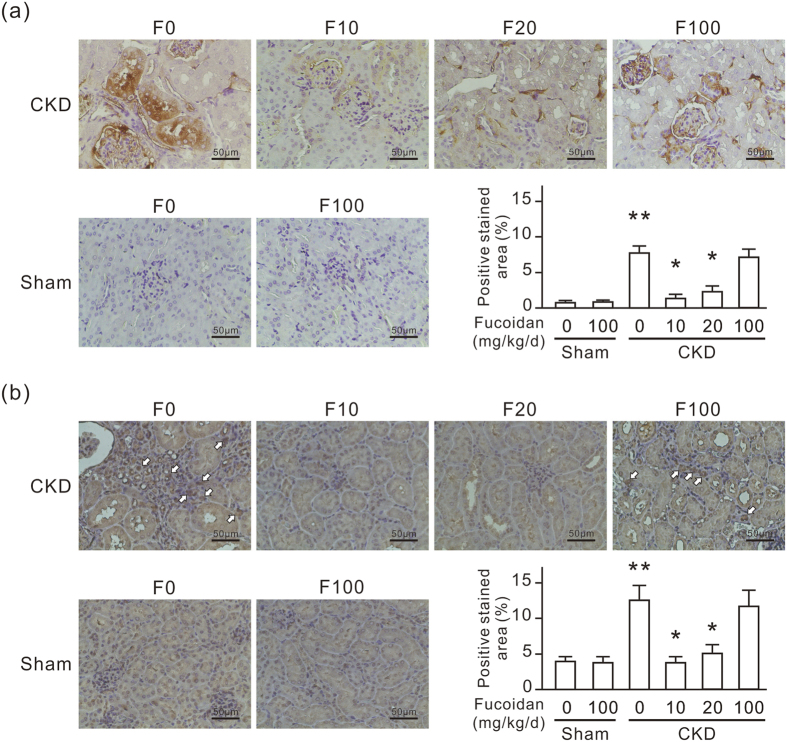
Oligo-fucoidan reduces the expression of renal fibronectin and αSMA in CKD mice. Six weeks after the surgery, the experimental mice were fed with or without oligo-fucoidan at 10 (F10), 20 (F20), or 100 (F100) mg/kg/d for 6 weeks. (**a**) Anti-fibronectin IHC-stained paraffin-embedded mouse kidney sections. (**b**) Anti-αSMA IHC-stained paraffin-embedded mouse kidney sections. The white arrows indicate fibroblasts stained by anti-αSMA. Scale bars = 50 μm. The positive stained areas in IHC stained slides are also presented in a bar chart. Data are presented as the mean ± SD from 6 mice in each group. *P < 0.05 vs. the CKD group without oligo-fucoidan. **P < 0.05 vs. the sham group without oligo-fucoidan.

Fibronectin was expressed in renal tubular cells and accumulated in the renal tubulointerstitial space in CKD mice as revealed by IHC (Fig. 3a). Dilated renal tubules also highly expressed αSMA, and fibroblasts accumulated in the renal tubulointerstitial space in CKD mice as revealed by αSMA IHC (Fig. 3b). Oligo-fucoidan treatment at a dose of 10 or 20 mg/kg/d for 6 weeks significantly reduced renal fibronectin and αSMA expression in CKD mice. This protective effect did not occur with the oligo-fucoidan treatment at 100 mg/kg/d. In the sham group, oligo-fucoidan at a dose of 100 mg/kg/d did not cause fibronectin and αSMA expression change in the kidneys.

### Oligo-fucoidan inhibits pressure-induced fibrotic responses in rat renal tubular cells

**Figure 4:**
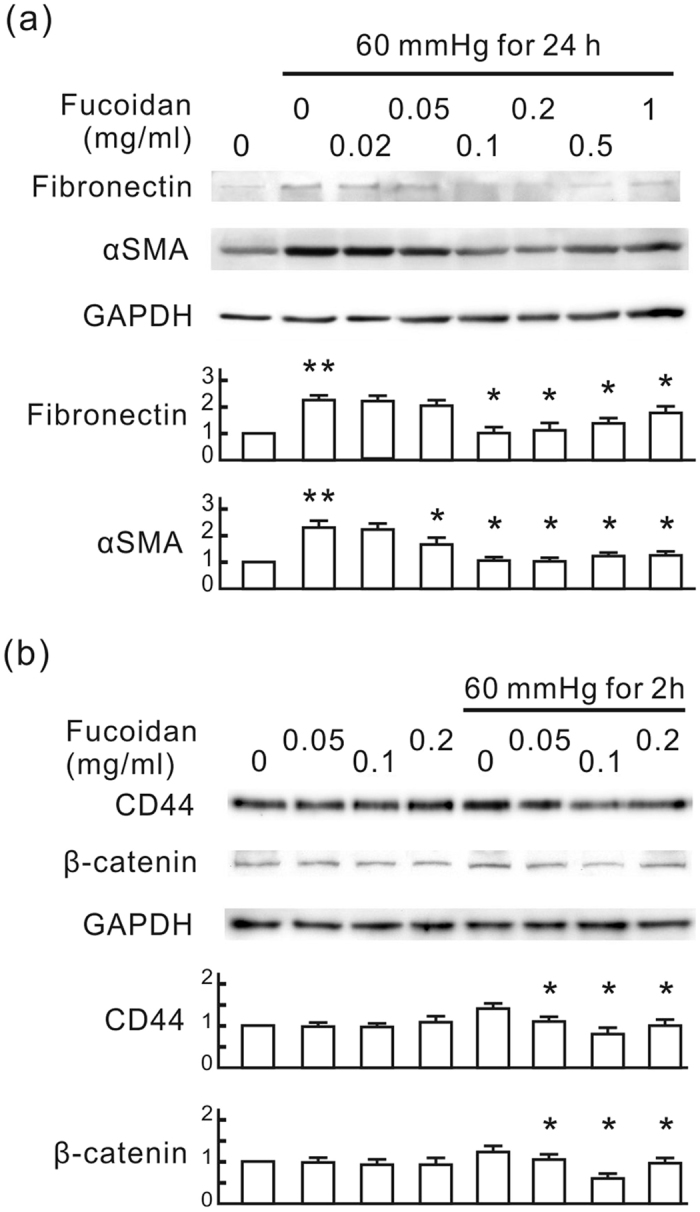
Oligo-fucoidan inhibits pressure-induced fibrotic responses in NRK-52E cells. NRK-52E cells were treated with oligo-fucoidan at different concentrations for 30 min, and then cultured in the pressure apparatus with 60 mmHg for the indicated periods. (**a**) Western blots of fibronectin and αSMA. (**b**) Western blots of CD44 and β-catenin. GAPDH was used as a loading control. The relative quantity of each band is also presented in a bar chart. Data are presented as the mean ± SD from 5 independent experiments. *P < 0.05 vs. the pressurized cells without oligo-fucoidan. **P < 0.05 vs. the control group.

**Figure 5:**
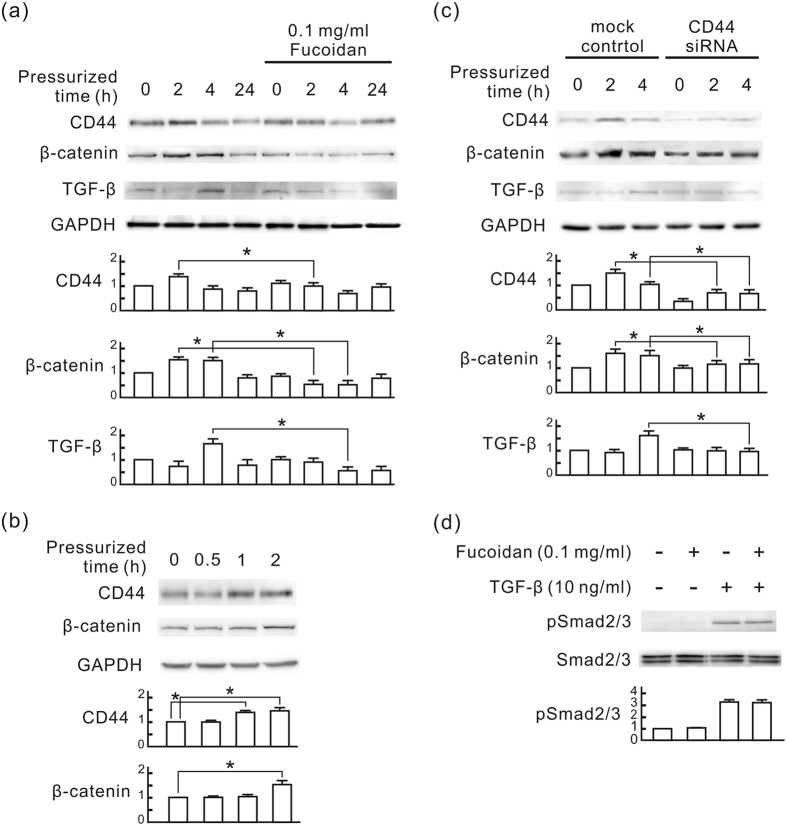
Oligo-fucoidan inhibits the CD44 signaling pathway in pressurized NRK-52E cells. (**a**) A time course study of expression of CD44, β-catenin, and TGF-β in pressurized NRK-52E cells. The cells were treated with 0.1 mg/ml oligo-fucoidan for 30 min, and then cultured in the pressure apparatus with 60 mmHg for the indicated periods. Western blot analysis was used to detect CD44, β-catenin, and TGF-β. GAPDH was used as a loading control. (**b**) The transient expression of CD44 and β-catenin in pressurized NRK-52E cells. The cells were cultured in the pressure apparatus with 60 mmHg for the indicated periods. Western blot analysis was used to detect CD44 and β-catenin. (**c**) The inhibitory effect of CD44 siRNA transfection on the expression of β-catenin and TGF-β. NRK-52E cells were transfected with CD44 siRNA or negative control siRNA (mock control) and then cultured in the pressure apparatus with 60 mmHg for the indicated periods. Western blot analysis was used to detect CD44, β-catenin, and TGF-β. (**d**) Independence of the anti-fibrotic effect of oligo-fucoidan from the TGF-β signaling pathway. NRK-52E cells were pretreated with 0.1 mg/ml oligo-fucoidan for 30 min, and then treated with 10 ng/ml TGF-β for 2 h. Western blot analysis was used to detect phospho-Smad2/3 and Smad2/3. The relative quantity of each band is also presented in a bar chart. Data are presented as the mean ± SD from 5 independent experiments. *P < 0.05.

To evaluate the renal protective mechanism of oligo-fucoidan, we applied 60 mmHg of pressure on rat renal tubular cells (NRK-52E) and monitored the influence of oligo-fucoidan on pressure-induced fibrotic responses *in vitro*. Following pressurization for 24 h, fibronectin and αSMA expression was significantly elevated in NRK-52E cells, which was inhibited by oligo-fucoidan (Fig. 4a). The inhibitory effect of oligo-fucoidan reached a maximum at the dosages of 0.1 and 0.2 mg/ml. Following pressurization for 2 h, CD44 and β-catenin expression was significantly elevated in NRK-52E cells, which was also inhibited by oligo-fucoidan (Fig. 4b). This inhibitory effect reached a maximum at the dosage of 0.1 mg/ml. In a time course study, CD44 was transiently induced at 1 and 2 h after pressurization. β-catenin was induced at 2 and 4 h, and TGF-β was induced at 4 h (Fig. 5a,b). This result implies that CD44 is an earlier event than the β-catenin and TGF-β signaling pathways in pressure-induced fibrotic responses in NRK-52E cells. We further reduced CD44 by siRNA transfection and evaluated the influence of CD44 on the expression of β-catenin and TGF-β in NRK-52E cells. As shown in Fig. 5c, CD44 knockdown significantly reduced both pressure-induced β-catenin and TGF-β at the corresponding point in time. We also found that oligo-fucoidan did not influence the TGF-β-induced Smad signaling pathway although it reduced pressure-induced TGF-β in NRK-52E cells (Fig. 5d). These results suggest that the inhibitory effect of oligo-fucoidan on fibrotic responses in NRK-52E cells may result from the reduction of pressure-induced CD44 expression.

### Oligo-fucoidan reduces the expression of CD44 in the kidneys of CKD mice

**Figure 6:**
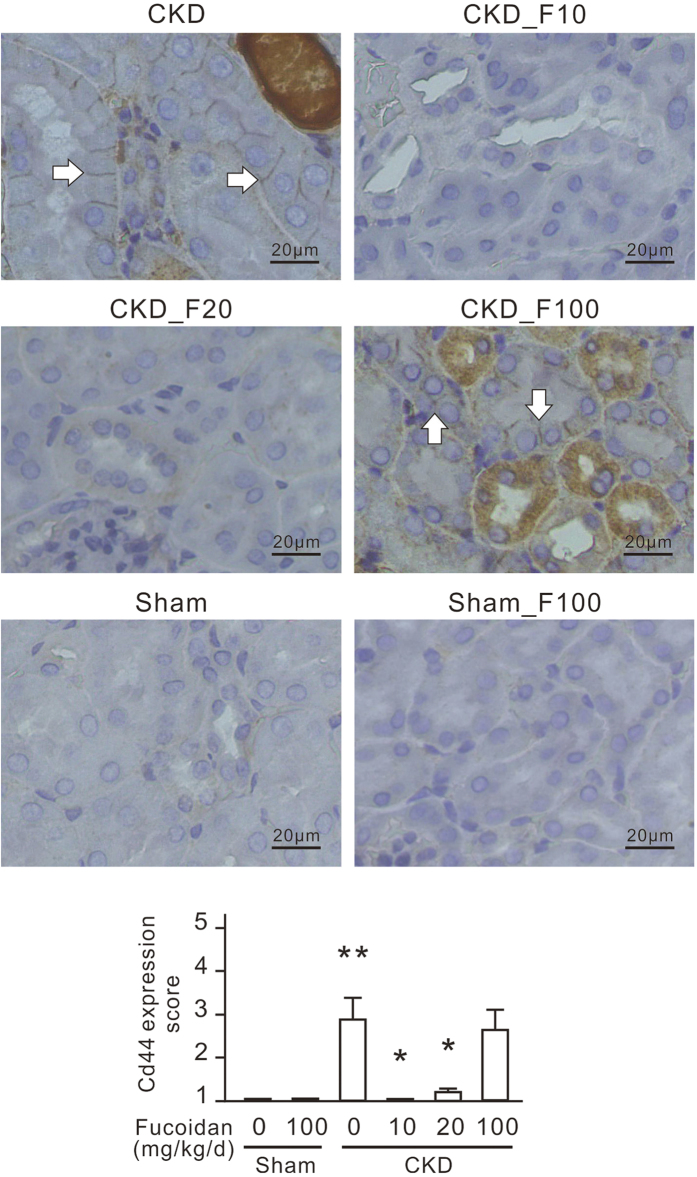
Oligo-fucoidan reduces CD44 expression in renal tubular cells in CKD mice. Six weeks after the surgery, the experimental mice were fed with or without oligo-fucoidan at 10 (F10), 20 (F20) or 100 (F100) mg/kg/d for 6 weeks, and then the kidneys were collected. Paraffin-embedded mouse kidney sections were stained by IHC with anti-CD44. The white arrows indicate the positive-stained signal. CD44 expression scores are shown as the means ± SD from 6 mice in each group. *P < 0.05 vs. the CKD group without oligo-fucoidan. **P < 0.05 vs. the sham group without oligo-fucoidan.

In CKD mice, CD44 expression increased in renal tubules with slight dilation and was located on the cell membrane, especially at the boundary between tubular cells (Fig. 6). Oligo-fucoidan treatment at a dose of 10 mg/kg/d inhibited CD44 induction in the dilated renal tubules in CKD mice. However, oligo-fucoidan treatment at a dose of 100 mg/kg/d failed to reduce the expression of CD44 in the kidneys of CKD mice.

### Oligo-fucoidan reduces the expression of renal β-catenin and TGF-β1 in CKD mice

**Figure 7:**
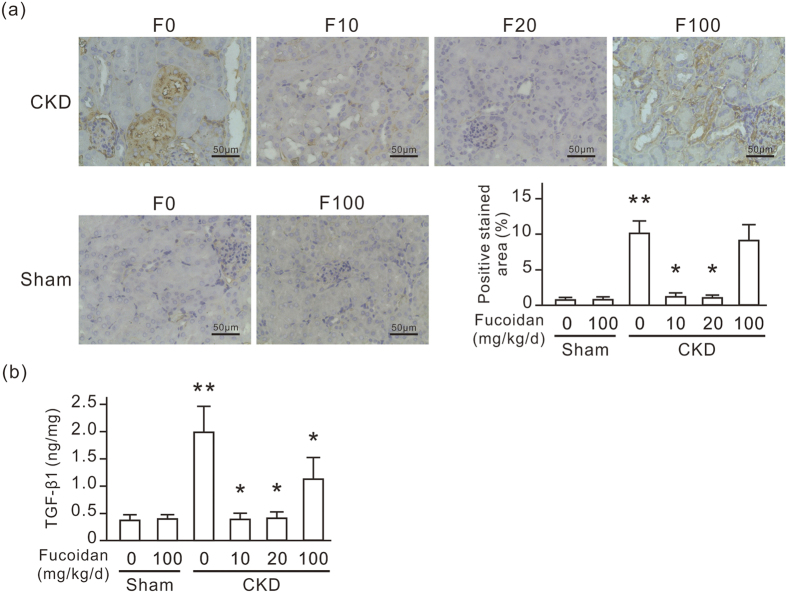
Oligo-fucoidan reduces the expression of renal β-catenin and TGF-β1 in CKD mice. Six weeks after the surgery, the experimental mice were fed with or without oligo-fucoidan at 10 (F10), 20 (F20), or 100 (F100) mg/kg/d for 6 weeks. (**a**) Anti-β-catenin IHC-stained paraffin-embedded mouse kidney sections. Scale bars = 50 μm. The positive stained areas in IHC stained slides are also presented in a bar chart. (**b**) TGF-β1 ELISA assay in kidney homogenate. Data are presented as the mean ± SD from 6 mice in each group. *P < 0.05 vs. the CKD group without oligo-fucoidan. **P < 0.05 vs. the sham group without oligo-fucoidan.

To confirm the connection between CD44 and β-catenin/TGF-β, we further monitored the expression of renal β-catenin and TGF-β1 in CKD mice. Dilated renal tubules highly expressed β-catenin in CKD mice as revealed by IHC (Fig. 7a). TGF-β1 level in the kidneys of CKD mice is also elevated (Fig. 7b). Oligo-fucoidan treatment at a dose of 10 or 20 mg/kg/d for 6 weeks significantly reduced renal β-catenin and TGF-β1 expression in CKD mice. This inhibitory effect on β-catenin did not occur with the oligo-fucoidan treatment at 100 mg/kg/d. However, the oligo-fucoidan treatment at 100 mg/kg/d moderately reduced TGF-β1 expression in CKD mice.

### Oligo-fucoidan interferes with the interaction between CD44 and its ligands

**Figure 8:**
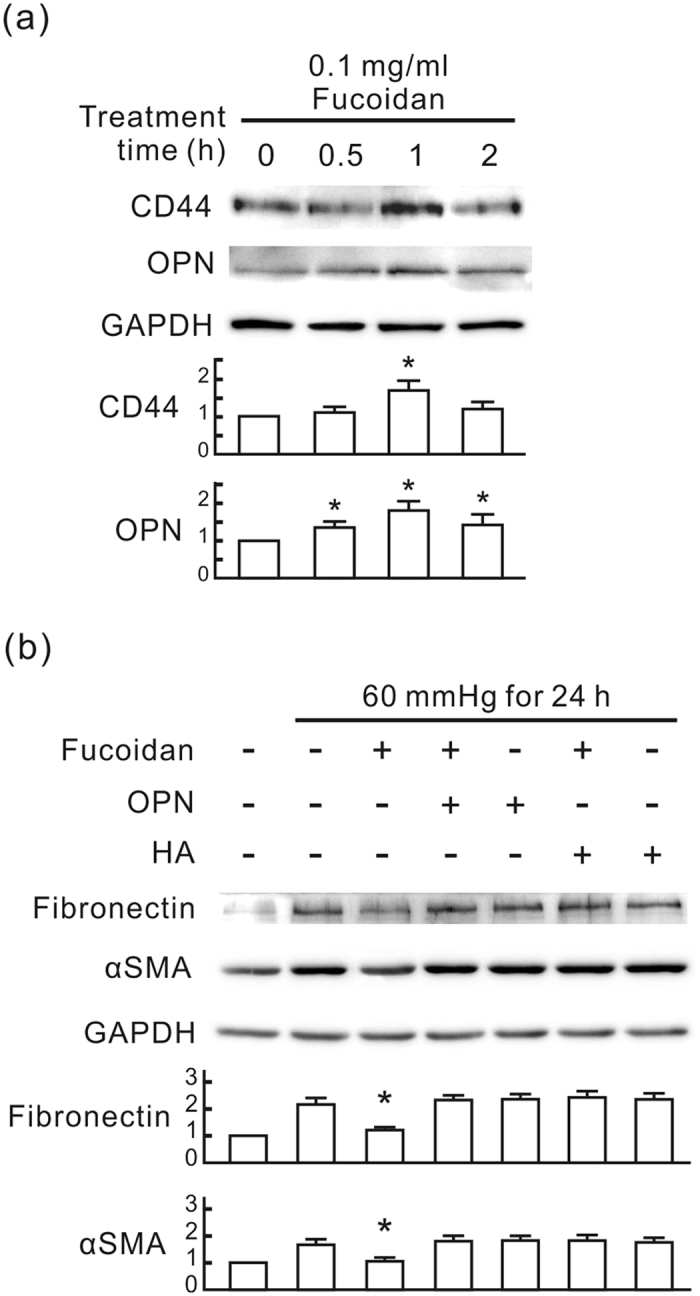
The anti-fibrotic effect of oligo-fucoidan is associated with the blockage of hyaluronic acid and osteopontin. (**a**) The transient induction of CD44 and osteopontin (OPN) by oligo-fucoidan in NRK-52E cells. The cells were treated with 0.1 mg/ml oligo-fucoidan for the indicated periods. Western blot analysis was used to detect CD44 and OPN. GAPDH was used as a loading control. The relative quantity of each band is also presented in a bar chart. Data are presented as mean ± SD from 5 independent experiments. *P < 0.05 vs. the group at 0 h. (**b**) The inhibitory effect of hyaluronan (HA) and osteopontin (OPN) on the anti-fibrotic effect of oligo-fucoidan. NRK-52E cells were pretreated with or without 0.1 mg/ml oligo-fucoidan for 30 min, treated with 65 μg/ml HA or 0.15 mg/ml OPN, and then cultured in the pressure apparatus with 60 mmHg for 24 h. Western blot analysis was used to detect fibronectin and αSMA. The relative quantity of each band is also presented in a bar chart. Data are presented as the mean ± SD from 5 independent experiments. *P < 0.05 vs. the pressurized cells without other treatment.

Our previous study showed that the blockage of the extracellular ligands of CD44, such as hyaluronic acid and osteopontin, transiently increases the expression of CD44 and osteopontin in NRK-52E cells^9^. Following oligo-fucoidan treatment at 0.1 mg/ml for 1 h, the expression of CD44 and osteopontin was also increased in NRK-52E cells (Fig. 8a). We applied hyaluronic acid or osteopontin to further identify the role of CD44 ligands in the anti-fibrotic effect of oligo-fucoidan. As shown in Fig. 8b, pressure-induced fibronectin and αSMA were reduced by oligo-fucoidan, which was completely recovered by additional hyaluronan or osteopontin. These results suggest that oligo-fucoidan interferes with the interaction between CD44 and its extracellular ligands in NRK-52E cells.

### Oligo-fucoidan reduces renal hyaluronic acid synthesis and osteopontin expression in CKD mice

**Figure 9:**
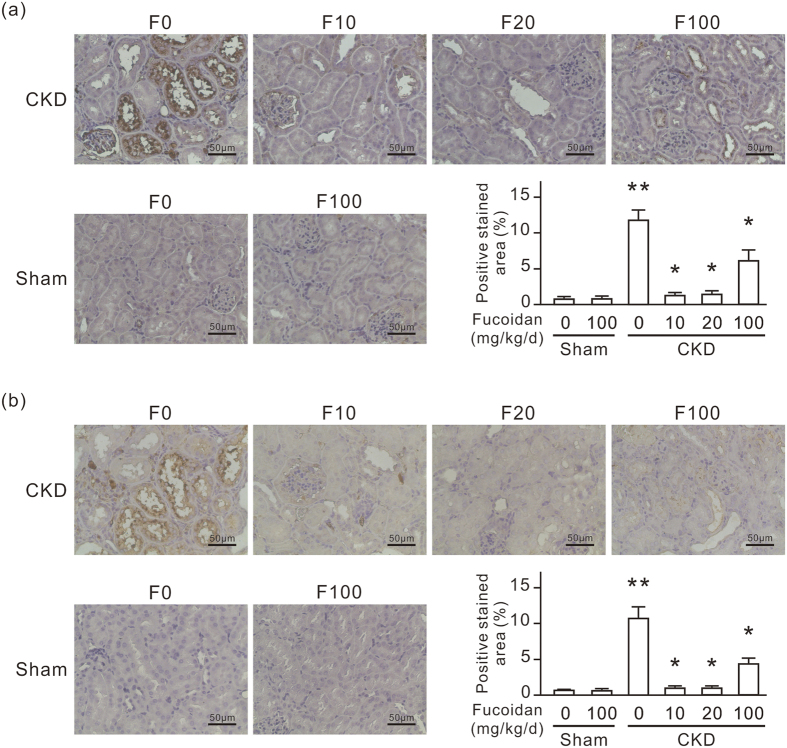
Oligo-fucoidan reduces the expression of renal hyaluronan and osteopontin in CKD mice. Six weeks after the surgery, the experimental mice were fed with or without oligo-fucoidan at 10 (F10), 20 (F20), or 100 (F100) mg/kg/d for 6 weeks. (**a**) Anti-hyaluronan IHC-stained paraffin-embedded mouse kidney sections. (**b**) Anti-osteopontin IHC-stained paraffin-embedded mouse kidney sections. Scale bars = 50 μm. The positive stained areas in IHC stained slides are also presented in a bar chart. Data are presented as the mean ± SD from 6 mice in each group. *P < 0.05 vs. the CKD group without oligo-fucoidan. **P < 0.05 vs. the sham group without oligo-fucoidan.

To check the influence of oligo-fucoidan on renal hyaluronic acid synthesis and osteopontin expression *in vivo*, we also monitored the levels of hyaluronic acid and osteopontin in the kidneys of CKD mice. The synthesis of hyaluronic acid and the expression of osteopontin were obviously increased in dilated renal tubules in CKD mice as revealed by IHC (Fig. 9). Oligo-fucoidan treatment at a dose of 10, 20, or 100 mg/kg/d for 6 weeks significantly reduced renal hyaluronic acid synthesis and osteopontin expression in CKD mice.

## Discussion

The animal study reported here shows that oligo-fucoidan inhibits renal tubulointerstitial fibrosis in CKD mice at a particular dose. Orally ingested oligo-fucoidan at a dose of 10 mg/kg/d effectively improved renal function and reduced tubulointerstitial fibrosis (Figs 1, 2 and 3). However, the dose of 100 mg/kg/d did not improve renal function and even slightly worsened renal histologic change in CKD mice. In animal studies, low-molecular weight fucoidan doses of 100 mg/kg/d or higher are often applied to studies of diseases such as cancers, diabetic retinopathy, renal acute injury, and osteoporosis, and exerts significant protective effects^25^,^26^,^27^,^28^. Our results revealed that the high oligo-fucoidan dose promoted renal function and increased the creatinine clearance rate (Fig. 1). This promoting effect on renal function may not be beneficial for kidneys with reduced nephrons in CKD mice. On the other hand, the results of the *in vitro* study also showed that the anti-fibrotic effect of oligo-fucoidan decreased at a high concentration (Fig. 4a). Therefore, the dosage should be carefully considered in the application of oligo-fucoidan to patients with CKD.

The first concern about an *in vitro* study with natural products is the absorption forms of the products. Recently, orally-ingested fucoidan was detected in serum in a rat model^29^. In that study, lower molecular weight fucoidan was observed in the urine, which indicates that low molecular weight fucoidan can be directly absorbed into the circulatory system via the digestive system. Additionally, intravenous-delivered fucoidan is accumulated in the kidney and excreted into the urine^30^. Therefore, orally-ingested fucoidan can affect renal tubular cells without any modification. The molecular weight of the oligo-fucoidan used in the present study was approximately 800 Da, which theoretically results in higher absorption efficiency and more influence on renal cells *in vivo*. Our results also show that the influence patterns of oligo-fucoidan on fibrotic responses *in vivo* and *in vitro* are almost identical, indicating that orally-ingested oligo-fucoidan directly affects renal tubular cells in mice.

Pressure is thought to be a major mechanism that induces renal fibrosis in ureteric obstruction^31^. Recent evidence suggests that the pressure force also contributes to the induction and progression of tubulointerstitial fibrogenesis in diabetic nephropathy^32^. The pressure force imparts mechanical strain to renal tubular epithelial cells resulting in tubular dilation and interstitial infiltrations and fibrosis. Pressure causes the secretion of pro-inflammatory mediators and profibrogenic cytokines in tubular epithelial cells, which is important in the renal fibrogenesis of obstructive nephropathy and diabetic nephropathy^33^,^34^. In the present study, we also found renal tubular dilation in CKD mice (Fig. 2). In the cellular study, the pressure force transiently up-regulated CD44 in NRK-52E cells (Fig. 5a,b). The expression of CD44 was also increased in the slightly dilated renal tubules (Fig. 6). The data of CD44 expression and tubular injury reveal that tubular CD44 expression levels are highly related with renal tubular injury levels (Figs 2c and 6). These results suggest that the pressure force plays a critical role in the induction and progression of tubulointerstitial fibrogenesis in CKD.

**Figure 10:**
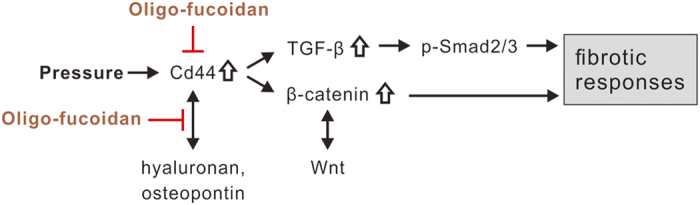
Schematic presentation of the inhibitory role of oligo-fucoidan in CD44-mediated fibrotic responses in renal tubular cells.

CD44 is a cell surface glycoprotein mediating cell adhesion and migration in a variety of pathophysiological processes, including tumor metastasis, wound healing, and inflammation^35^. The two main ligands for CD44 are hyaluronan (HA) and osteopontin (OPN)^36^,^37^. HA is a water-soluble glycosaminoglycan and has important structural functions in the extracellular matrix of all tissues. Increased HA causes an aggressive phenotype leading to severe lung fibrosis after bleomycin-induced injury^38^. OPN is an immobilized matrix phosphorylated glycoprotein^39^ that plays a key role in diabetic nephropathy^40^. The transfection of OPN siRNA *in vivo* inhibited the progression of chronic allograft nephropathy^41^. A previous study revealed that the blockage of extracellular HA and OPN by pep-1 and OPN neutralizing antibody, respectively, prevents pressure-induced fibrotic responses in renal tubular cells, but mildly increases the expression of CD44 and OPN at the early stage of the treatment^9^. Our results showed that the oligo-fucoidan treatment also increased the expression of CD44 and OPN at the early stage of treatment in NRK-52E cells (Fig. 8a), which implies that oligo-fucoidan interferes with the interaction between CD44 and HA/OPN. Additional HA or OPN, as expected, significantly reduced the anti-fibrotic effect of oligo-fucoidan in NRK-52E cells (Fig. 8b). Moreover, oligo-fucoidan treatment also reduced renal HA and OPN expression level in CKD mice (Fig. 9). Besides the inhibitory effect on CD44 expression, we suggest that interference with the interaction between CD44 and its extracellular ligands is also an important mechanism of the renal protective effect of oligo-fucoidan in CKD mice (Fig. 10).

In summary, our current results demonstrate that orally administered oligo-fucoidan at a low dose significantly improves renal function and reduces tubulointerstitial fibrosis in CKD mice. We found that oligo-fucoidan inhibited pressure-induced CD44 *in vivo* and *in vitro*. The inhibition of transient CD44 increase reduced the expression of β-catenin and TGF-β, which further inhibited pressure-induced fibrotic responses in NRK-52E cells. The *in vitro* study with additional HA and OPN reveals that interference in the interaction between CD44 and its extracellular ligands is one mechanism underlying oligo-fucoidan-mediated inhibition of fibrotic responses in renal tubular cells. Therefore, our current findings indicate that oligo-fucoidan has potential as a therapeutic intervention in controlling renal tubulointerstitial fibrosis in CKD patients.

## Methods

### Materials

The oligo-fucoidan powder was obtained from Hi-Q Marine Biotech International (Taiwan) and prepared by enzyme hydrolysis of original fucoidan. In brief, 5 g of fucoidan from *Sargassum hemiphyllum* was suspended in 125 mL distilled water at 55 °C with at 700 rpm stirring speed followed by addition of glycolytic enzyme at a concentration of 1 mg/g fucoidan for 6 h. After centrifugation at 10,000 × g for 20 min at 4 °C, the supernatants were passed through a 30 kDa molecular weight cut-off membrane (ProStream™ PP, TangenX Technology Co., Boston, MA, USA) and the filtrate was further passed through a 1 kDa molecular weight cut-off membrane to obtain the oligo-fucoidan with the average molecular weight of 800 Da. Oligo-fucoidan was dissolved in double-distilled H_2_O and filtered using 0.22-μm sterile filters (Merck Millipore, Darmstadt, Germany). Hyaluronan (molecular weight 0.6–1.1 MDa) and all other chemicals of reagent grade were obtained from Sigma (St. Louis, MO, USA). Recombinant mouse osteopontin and anti-osteopontin (AF808) were obtained from R&D Systems (Minneapolis, MN, USA). Antibodies against fibronectin (sc-9068) and Smad2/3 (sc-8332) were purchased from Santa Cruz Biotechnology (Dallas, TX, USA). Antibodies against αSMA (4968 s), β-catenin (8480 s), CD44 (3570 s), p-Smad2/3 (8828 s), and TGF-β (3711 s) were obtained from Cell Signaling Technology (Danvers, MA, USA).

### Cell culture

Rat renal tubular cells (NRK-52E) were purchased from the Food Industry Research and Development Institute (Taiwan). The cells were cultured in DMEM supplemented with an antibiotic/antifungal solution and 17% foetal bovine serum and were grown until the monolayer became confluent. The cultured cells were then cultured in serum-free medium and incubated overnight before the experiment. Cells were used between the 20^th^ and 30^th^ passage counted from the day of receipt. To study the effects of pressure on cells *in vitro*, we established a pressure apparatus as previously reported^7^. The entire system was placed in a CO_2_ incubator to maintain 37 °C and 95% humidity.

### Mouse CKD model and oligo-fucoidan treatment

All animal experiments were approved by the Taipei Medical University Committee of Experimental Animal Care and Use (approval No. LAC-2014-0292), and performed in accordance with relevant guidelines and regulations. Male 8-week-old 129S1/SvImJ mice were obtained from Lasco Technology (Taiwan). The CKD model was generated in 129S1/SvImJ mice at 9 weeks of age (weight 21–23 g) by uninephrectomy plus ischemic-reperfusion injury to the contralateral kidney as previously reported^42^. Sham-operated mice underwent laparotomy and manual manipulation of the kidneys. The mice were anesthetized by an intraperitoneal injection of tribromoethanol (250 mg/kg) before the operation. Six weeks after the surgery, we fed the experimental mice oligo-fucoidan for 6 weeks. The experimental mice were divided into 6 groups with n = 6: (1) Sham group; (2) CKD group; (3) Sham group fed oligo-fucoidan (100 mg/kg/d); (4) CKD group fed oligo-fucoidan (10 mg/kg/d); (5) CKD group fed oligo-fucoidan (20 mg/kg/d); (6) CKD group fed oligo-fucoidan (100 mg/kg/d).

For the metabolic study, the blood and the 24-h urine samples were analyzed from each mouse by a biochemical analyzer to detect renal function. We recorded the creatinine clearance rate (CCr) (calculated using the formula: CCr = (urine creatinine × urine volume)/(plasma creatinine × 1440 min)) of each mouse. After sacrifice, changes in renal morphology were examined in formalin-fixed, 2-μm paraffin sections by periodic acid-Schiff (PAS) staining and immunohistochemistry staining (IHC).

### Immunohistochemistry staining

For immunohistochemistry staining, kidney tissues fixed with 4% buffered paraformaldehyde were embedded in paraffin and 2 μm sections were prepared. The sections were stained with UltraVision Quanto Detection System HRP DAB kits (Thermo Scientific, Fremont, CA, USA) and hybridized with the anti-fibronectin antibody according to the manufacturer’s instructions.

### Histomorphometric analysis

After PAS, Masson’s trichrome and IHC staining, we collected 10 images of a kidney from each mouse at 200x magnification. Areas of positive staining for particular antibodies in the entire cortical tubulointerstitium were examined using the quantitative software Image-Pro Plus 6 (Media Cybernetics, Rockville, MD, USA) as described in the previous study^43^. For PAS staining intensity, we used two color segmentations; one recognized PAS-positive magenta areas and the other light pink background areas. PAS-stained area percentage was calculated as the number of magenta pixels divided by the sum of magenta and light pink pixels. For Masson’s trichrome staining intensity, we used two color segmentations; one recognized blue areas and the other red areas. Blue area percentage was calculated as the number of blue pixels divided by the sum of blue and red pixels. CD44 expression scoring was made in the cortex using CD44-IHC-stained renal tissue sections. The percentage of positive-stained tubules was estimated using a 5-point scale in 10 randomly chosen, nonoverlapping fields (200x magnification). CD44 expression scores were graded on a scale from 0 to 5: 0 = no involvement of total tubules; 1 = <10% involvement of total tubules; 2 = 10–25% involvement of total tubules; 3 = 25–50% involvement of total tubules; 4 = 50–75% involvement of total tubules; and 5 = >75% involvement of total tubules.

### Histopathological scoring

Tubular injury scoring was made in the cortex using PAS-stained renal tissue sections. The percentage of damaged tubules was estimated using a 5-point scale according to the following criteria: tubular dilatation, cast deposition, brush border loss, and necrosis in 10 randomly chosen, nonoverlapping fields (200x magnification)^44^. Lesions were graded on a scale from 0 to 5: 0 = normal kidney; 1 = <10% involvement; 2 = 10–25% involvement; 3 = 25–50% involvement; 4 = 50–75% involvement; and 5 = >75% involvement.

### Western blot analysis

A total of 15 μg of NRK-52E protein lysate was applied to each lane and analyzed by western blotting. Relative levels of the protein bands were quantified from five independent experiments by Quantiscan software (Biosoft, Cambridge, United Kingdom). The original blots are shown in the Supplementary Information.

### Short interfering (si)RNA transfection of CD44

We purchased rat CD44 siRNA (4390771) from Thermo Scientific. Cells were grown to 70% confluence, and the siRNAs were transfected using the Lipofectamine 2000 transfection reagent (Thermo Scientific) according to the manufacturer’s instructions. The transfection efficiency for CD44 siRNA was greater than 70%. We replaced the culture medium after 24 h for pressurization and western blot assays.

### TGF-β1 ELISA assay

The collected kidneys were cut in small pieces, frozen in liquid nitrogen, and subsequently stored at −70 °C until further usage. To obtain tissue lysates, approximately 30 to 50 mg of tissues were homogenized in 500 μl of lysis buffer (50 mM Tris, pH 7.5, 1% Nonidet P-40, 0.5% sodium deoxycholate, 150 mM NaCl, and protease inhibitors) on ice. Samples were centrifuged at 13,000 rpm for 20 minutes at 4 °C. Clear supernatant was collected and analyzed using TGF-β1 ELISA kit (R&D Systems, Minneapolis, MN, USA) according to the manufacturer’s instructions.

### Statistical tests

Significant differences between two groups were determined using Mann-Whitney test. Differences were considered significant for p values < 0.05.

## Additional Information

**How to cite this article**: Chen, C.-H. *et al*. Oligo-fucoidan prevents renal tubulointerstitial fibrosis by inhibiting the CD44 signal pathway. *Sci. Rep.* **7**, 40183; doi: 10.1038/srep40183 (2017).

**Publisher's note:** Springer Nature remains neutral with regard to jurisdictional claims in published maps and institutional affiliations.

## Supplementary Material

Supplementary Information
