# Supplementary material for: Oligo-fucoidan prevents renal tubulointerstitial fibrosis by inhibiting the CD44 signal pathway
Source: Sci Rep. 2017 Jan 18;7:40183. doi: 10.1038/srep40183 (PMC5241801; doi:10.1038/srep40183)
Supplement: Supplementary Information [file srep40183-s1.pdf]

Oligo-fucoidan prevents renal tubulointerstitial fibrosis by inhibiting the CD44 signal pathway

Cheng-Hsien Chen[1,2,3,4], Yuh-Mou Sue[1], Chung-Yi Cheng[1], Yen-Cheng Chen[1], Chung-Te Liu[1], Yung-Ho Hsu[2,3], Pai-An Hwang[5], Nai-Jen Huang[1], Tso-Hsiao Chen[1,2,*]

[1] Division of Nephrology, Department of Internal Medicine, Wan Fang Hospital, Taipei Medical University, Taipei, Taiwan.

[2] Department of Internal Medicine, School of Medicine, College of Medicine, Taipei Medical University, Taipei, Taiwan.

[3] Division of Nephrology, Department of Internal Medicine, Shuang Ho Hospital, Taipei Medical University, New Taipei City, Taiwan.

[4] School of Medicine, National Defense Medical Center, Taipei, Taiwan.

[5] Department of Bioscience and Biotechnology, National Taiwan Ocean University, Taiwan.

***Correspondence:**

Tso-Hsiao Chen

Email: tsohsiao@yahoo.com.tw, Tel: 886-2-29307930 ext. 2896

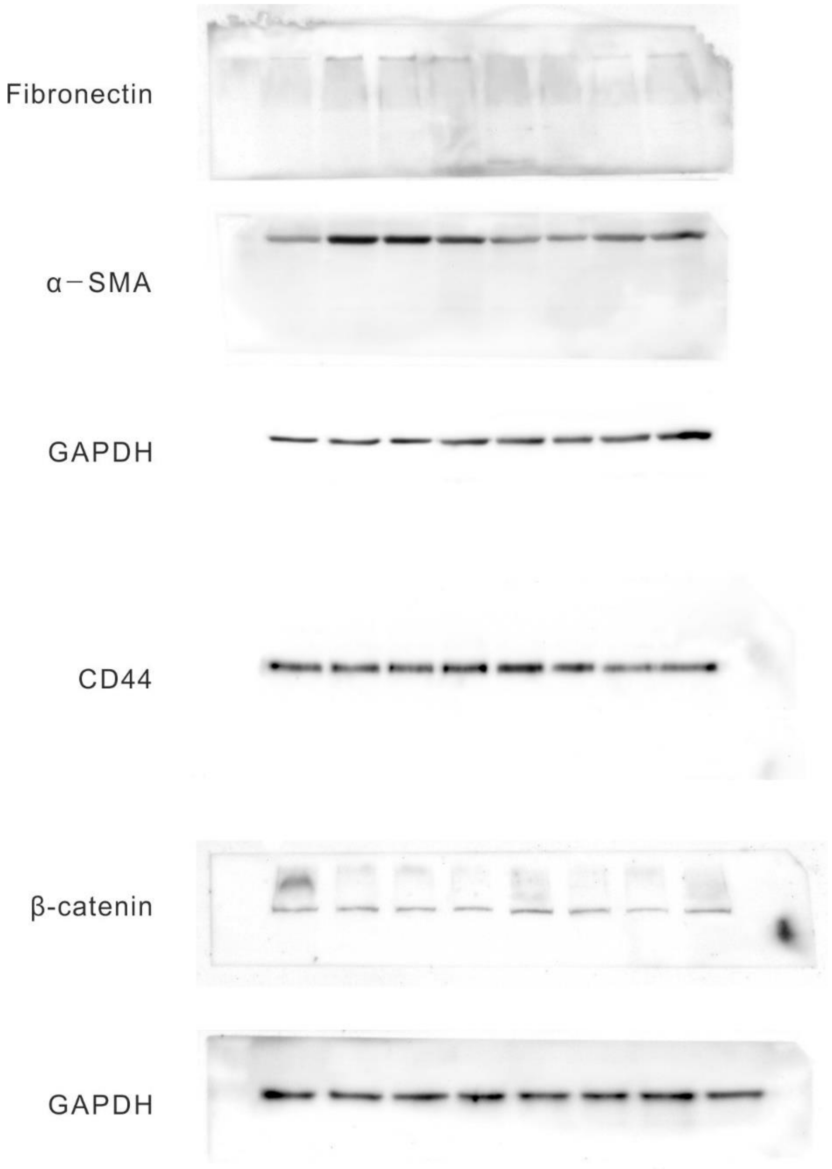

Figure S1. The original blots in Figure 4.

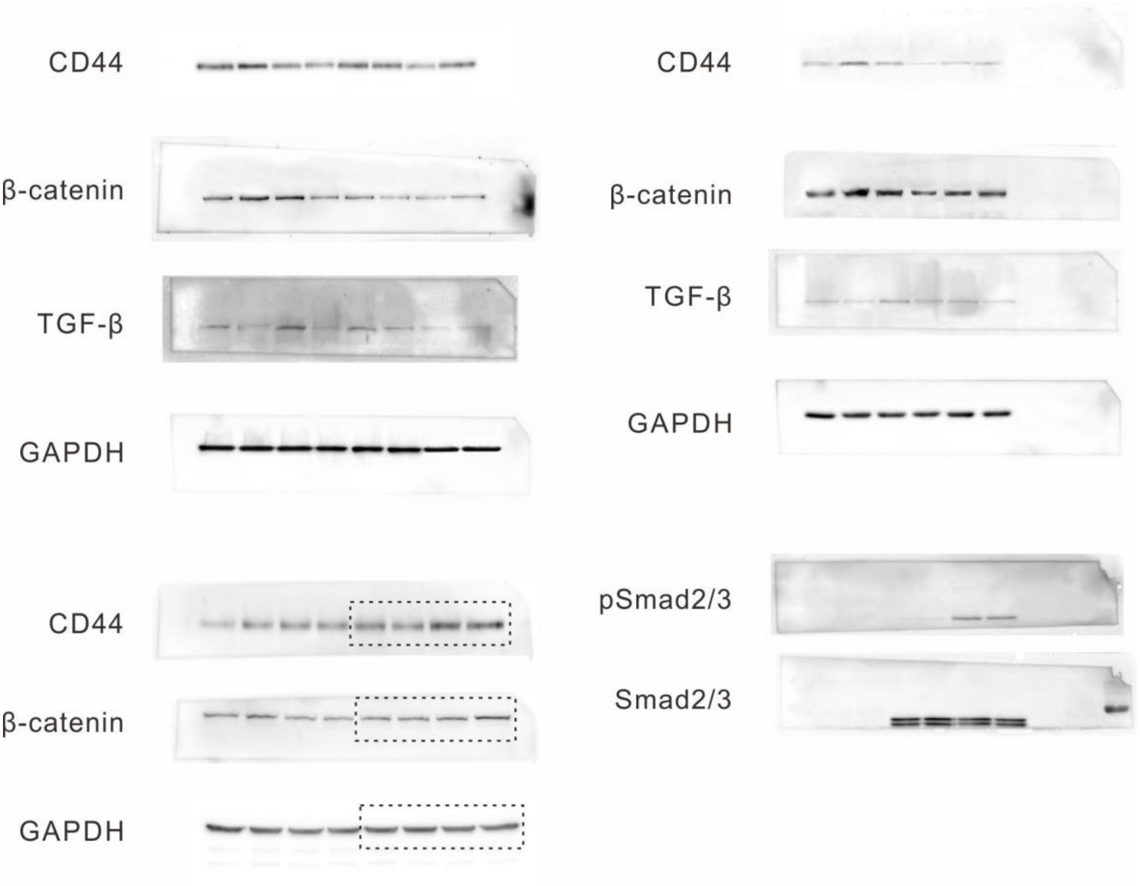

Figure S2. The original blots in Figure 5.

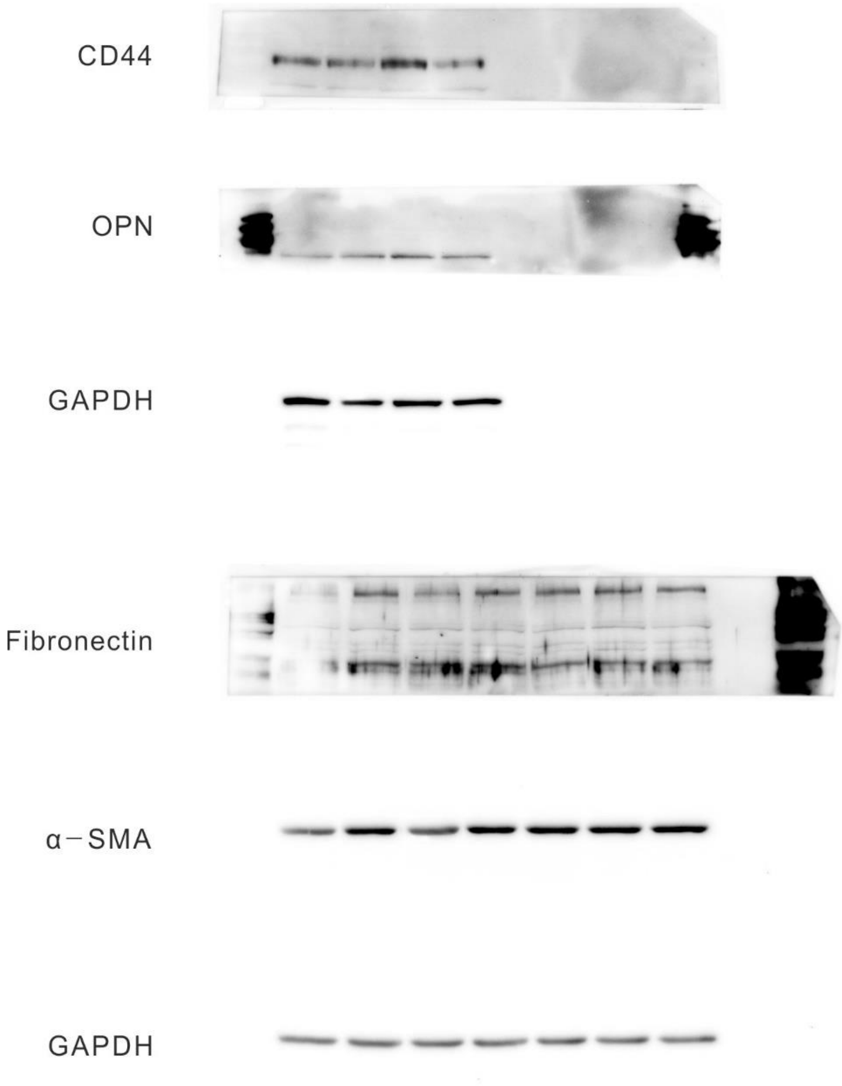

Figure S3. The original blots in Figure 8.
